# Host–microbiota interaction induces bi-phasic inflammation and glucose intolerance in mice

**DOI:** 10.1016/j.molmet.2017.08.016

**Published:** 2017-09-21

**Authors:** Antonio Molinaro, Robert Caesar, Louise Mannerås Holm, Valentina Tremaroli, Patrice D. Cani, Fredrik Bäckhed

**Affiliations:** 1 Wallenberg Laboratory, Department of Molecular and Clinical Medicine and Sahlgrenska Center for Cardiovascular and Metabolic Research, University of Gothenburg, 413 45 Gothenburg, Sweden; 2 WELBIO – Walloon Excellence in Life Sciences and BIOtechnology, Louvain Drug Research Institute, Université catholique de Louvain, Brussels, Belgium; 3 Metabolism and Nutrition Research Group, Louvain Drug Research Institute, Université catholique de Louvain, Brussels, Belgium; 4 Novo Nordisk Foundation Center for Basic Metabolic Research and Section for Metabolic Receptology and Enteroendocrinology, Faculty of Health Sciences, University of Copenhagen, Copenhagen 2200, Denmark

**Keywords:** Microbiota, Germ-free, Antibiotic, Colonization, Adiposity, Glucose metabolism

## Abstract

**Objective:**

Gut microbiota modulates adiposity and glucose metabolism in humans and mice. Here we investigated how colonization of germ-free (GF) mice affects kinetics of adiposity and glucose metabolism.

**Methods:**

Adiposity and glucose metabolism were evaluated at different time points in ex-GF and antibiotic treated mice after colonization with gut microbiota from a conventionally raised (CONV-R) mouse. Mouse physiology, microbiome configuration, serum cytokine levels, and gene expression for inflammatory markers were performed in different tissues.

**Results:**

Colonization resulted in a bi-phasic glucose impairment: the first phase occurring within 3 days of colonization (*early phase*) and the second 14–28 days after colonization (*delayed phase*). The *early phase* co-occurred with an inflammatory response and was independent of adiposity, while the *delayed phase* was mostly ascribed to adipose tissue expansion and inflammation. Importantly, re-colonization of antibiotic treated mice displays only the *delayed phase* of glucose impairment and adiposity, suggesting that the *early phase* may be unique to colonization of the immature GF mice gut.

**Conclusions:**

Our results provide new insights on host–microbiota interaction during colonization of GF mice and the resulting effects on adiposity and glucose metabolism in a time resolved fashion.

## Highlights

• Inflammation affects glucose tolerance in early phase of colonization of GF mice.
• Adiposity affects glucose tolerance in *delayed phase* of colonization of GF mice.
• Colonization of antibiotics treated mice show only the delayed phase of glucose impairment.

## 1 Introduction

Bacterial colonization of the mammalian gut starts at birth [1]. It is a complex process that undergoes several phases of maturation that involves several factors and results in the development of the immune system [1], [2], [3]. The gut microbiota has emerged as an important factor regulating host physiology and metabolism, in particular glucose metabolism and adiposity [4]. Several studies in humans support the central role of gut microbiota on adiposity and glucose metabolism, showing an altered microbiota composition in subjects with obesity and type 2 diabetes [5], [6], [7]. However, the underlying mechanisms are just starting to be unraveled. The microbial cell membrane component lipopolysaccharide (LPS) induces low-level inflammation and influences adiposity and glucose tolerance in both mice and humans [8], [9], [10], providing a putative mechanism for the role of microbial-derived compounds in the development of the metabolic syndrome. Thus, modulation of gut microbiota and of microbially-produced compounds in mice and humans has been suggested to improve glucose metabolism, indicating a possible causative role of bacteria in the regulation of the host metabolic status [11], [12], [13], [14].

Germ free (GF) mice can be used to study the effect of microbiota–host interaction on metabolism [15]. GF mice have better glucose tolerance and lower adiposity than conventionally raised (CONV-R) mice that are colonized with a normal microbiota at birth [16]. Colonization of GF mice at adulthood with a normal microbiota results in impairment of glucose metabolism and fat deposition [10], [16], [17]. In agreement with these results, ablation of the gut microbiota in CONV-R mice reduces adiposity and improves glucose metabolism [18], [19].

Colonization of GF mice allows studying the kinetics of host–microbiota interaction and provides unique opportunities to investigate the key events following colonization [15], [20]. This model has been used to study the development of the immune system [20], [21], [22] and have shown that gut microbiota colonization is a rapid and dynamic process, characterized by the appearance of a low-diversity microbiota around 2 days post-colonization, followed by the succession within 2–3 weeks of a more complex community resembling the initial inoculum [20], [21], [23], [24]. Several studies have provided mechanistic evidence for how the development of a gut microbiota can result into the establishment of an immune homeostasis promoting the maturation of the immune system in the host [20], [22]. Besides the effects on the immune system, the development of a gut microbiota also influences the regulation of host metabolic genes in the intestine, thus indicating a possible link between gut microbiota, the immune system, and metabolism [23].

Although it is known that colonization of GF mice affects glucose metabolism and adiposity, there is limited knowledge about how the development of microbiota and its interaction with the host affects adiposity and glucose metabolism in a time-resolved fashion [10]. Here we perform a time-resolved study of how microbial colonization of GF mice affects adiposity and glucose metabolism.

## 2 Materials and methods

### 2.1 Mice experiments

GF male Swiss Webster mice, 10–12 weeks old, were maintained in flexible film isolators under a strict 12 h light cycle. GF status was verified regularly by anaerobic culturing in addition to PCR for bacterial 16S rRNA gene. All mouse experiments were performed on autoclaved chow diet (Labdiet) ad libitum.

Colonization and re-colonization experiments were performed in 4 h fasted mice. The cecum content of an age-, strain-, and sex-matched mouse was dissolved in 5 ml of buffer (PBS, 0.2 g/L Na_2_S and 0.5 g/L cysteine as reducing agents). 200 μl of solution were gavage in 4 h fasted GF/antibiotic treated mice. For antibiotic treatment, mice were treated with a combination of 4 different antibiotics in the drinking water (1 g/L ampicillin, 1 g/L metronidazole, 0.5 g/L vancomycin and 0.5 g/L neomycin, Sigma, St Louis, MO) for 1 week. Antibiotic solution was changed every 48 h and kept in light protected bottles. Epididymal white adipose tissue, liver, small intestine, and serum were harvested 4 h after fasting.

### 2.2 MRI, insulin and cytokines measurement

MRI, insulin, and glucose tolerance tests and measurements of insulin levels were performed as earlier described [10]. Briefly, insulin and glucose tolerance tests were performed by injecting insulin (0.75 U/kg body weight) or glucose (2 g/kg body weight), respectively, intraperitoneally after a 4 h fast. Tail blood samples were collected at 0, 15, 30, 60, 90, and 120 min and blood glucose levels were determined using a glucose meter (Accu Check Aviva, Roche). Insulin and cytokines serum levels were measured with kits from Meso Scale (Gaithersburg, Maryland, USA), and Crystal Chem (Downers Grove, Illinois, USA), respectively, according to the manufacturers' protocols.

### 2.3 Immunohistochemistry of EWAT and liver

Paraffin-embedded epididymal EWAT and liver sections (7 μm) were processed as previously described [10]. Briefly, slides were deparaffinized and processed for antigen retrieval with a 2100 Retriever using 13 DIVA solution and Hot Rinse (HistoLab Products AB, Gothenburg, Sweden). Endogenous peroxidase activity was quenched with 30% H_2_O_2_ for 30 min. Blocking in 5% rabbit serum, 1% bovine serum albumin, and 0.1% Triton X-100 at room temperature for 30 min. EWAT macrophages were stained with MAC-2/galectin-3 antibody (diluted 1:500 in blocking buffer overnight at 4 °C), and then detected with a biotinylated anti-rat (10 mg/ml) antibody. Immune complexes were detected by Vulcan Red reagent (Vector Laboratories, USA), according to the manufacturer's instructions. Crown-like structures were counted in 15–40 mm^2^ of histological sections per tissue. For the liver staining, F4/80 antibody was diluted 1:500 and incubated in blocking buffer 2 h at room temperature and then detected with a HRP anti-rat antibody diluted 1:100. Immune complexes were detected by Vectastain ABC kit reagents (Vectorlab, USA) according to the manufacturer's instructions counterstained with hematoxylin and quantitated by densitometric analysis using Biopix iQ software (version 2.1.3; Biopix, Sweden). List of antibodies is reported in online Supplementary Table 3.

### 2.4 Flow cytometry analysis

Adipose tissue was minced thoroughly and suspended in digestion solution (PBS, 2% bovine serum albumin, 20 mg collagenase type II, Sigma Aldrich, St Louis, Missouri, USA). Tissue digestion was performed at 37 °C using a shaker at 120 rpm for 40 min. The fat layer was removed, and cells were passed thought a 70 μm cell strainer. Cells were centrifuged (500 g, 4 °C, 5 min), washed in PBS selection buffer (PBS, 2 mM EDTA, and 2% BSA), and subsequently treated with ACK solution (NH_4_Cl 150 mM, KHCO_3_ 10 mM, and Na_2_EDTA 0.1 mM) for 7 min to remove red blood cells. Cells were washed and suspended in PBS selection buffer with BD Fc Block for 5 min, followed by incubation with antibody cocktails (CD11b/CD11c/Gr1 or CD3/CD4/CD8, respectively, see Supplementary Table 3 for details). After washing, cells were analyzed using an Accuri C6 flow cytometer (Accuri Cytometers, Ann Arbor, Michigan, USA). Data was processed using FlowJo 10 analysis software (FlowJo LLC, Ashland, Oregon, USA). A similar protocol was used for spleen and blood samples without the collagen digestion steps.

### 2.5 LPS measurement

Blood was collected from the mouse portal vein using a pyrogen free syringe/needle, and plasma was immediately isolated and frozen in liquid nitrogen. LPS concentration was measured using Endosafe-MCS (Charles River, Lyon, France) based on the limulus amoebocyte lysate (LAL) kinetic chromogenic methodology that measures color intensity directly related to the endotoxin concentration in a sample. Plasma was diluted with endotoxin-free buffer to minimize interferences in the reaction (inhibition or enhancement) and heated for 15 min at 70 °C. Each sample was diluted with endotoxin-free LAL reagent water (Charles River) and treated in duplicate, and two spikes for each sample were included in the determination [25].

### 2.6 Quantitative RT-PCR

Quantitative RT-PCR RNA was isolated using RNeasy kit with on-column DNase treatment (Qiagen, Hilden, Germany). cDNA templates were synthesized from total RNAs using the high-capacity cDNA reverse transcription kit (Applied Biosystems, Foster City, California, USA) according to the manufacturer's instructions. qRT-PCR assays were performed in 10 μl reactions containing 8 μl SYBR Green Master Mix buffer (Thermo Scientific, Waltham, Massachusetts, USA), and 2 μl of 900 nM gene-specific primers (300 nM primer concentrations were used to assess L32 transcripts). Gene expression data were normalized to the ribosomal protein L32. Primer sequences are reported in online Supplementary Table 4.

### 2.7 Liver lipid analysis

Snap frozen liver tissues were homogenized in 1.5 ml Chloroform/methanol (2:1 v/v). Lipids were measured using Infinity Triglycerides kit (Thermo Fischer Scientific).

### 2.8 Extraction of fecal genomic DNA and profiling of the fecal microbiota

Fresh fecal pellets for extraction of fecal genomic DNA were collected from 5 mice after 1, 3, 7, 14, and 28 days of colonization. Total fecal genomic DNA was extracted from one fecal pellet using a repeated bead beating method based on a protocol previously described [26]. Briefly, fecal pellets were placed in Lysing Matrix E tubes (MP Biomedicals) and extracted twice in lysis buffer (4% w/v SDS; 500 mmol/L NaCl; 50 mmol/L EDTA; 50 mmol/L Tris·HCl; pH 8) with bead beating at 5.0 m/s for 60 s in a FastPrep^®^-24 Instrument (MP Biomedicals). After each bead-beating cycle, samples were heated at 85 °C for 15 min and then centrifuged at full speed for 5 min at 4 °C. Supernatants from the two extractions were pooled and the DNA was first precipitated with isopropanol and then purified using the QIAamp DNA Mini kit (QIAGEN). Samples were eluted in 120 μL of AE buffer (10 mmol/L Tris·Cl; 0.5 mmol/L EDTA; pH 9.0).

The fecal microbiota was profiled by sequencing the V4 region of the 16S rRNA gene on an Illumina MiSeq instrument (Illumina RTA v1.17.28; MCS v2.5) with 515F and 806R primers designed for dual indexing [27] and the V2 Illumina kit (2 × 250 bp paired-end reads). 16S rRNA genes were amplified in duplicate reactions in volumes of 25 μl containing 1× Five Prime Hot Master Mix (5 PRIME GmbH), 200 nM of each primer, 0.4 mg/ml BSA, 5% DMSO, and 20 ng of total fecal genomic DNA. PCR was carried out under the following conditions: initial denaturation for 3 min at 94 °C, followed by 25 cycles of denaturation for 45 s at 94 °C, annealing for 60 s at 52 °C, and elongation for 90 s at 72 °C, and a final elongation step for 10 min at 72 °C. Duplicates were combined, purified with the NucleoSpin Gel and PCR Clean-up kit (Macherey–Nagel), and quantified using the Quant-iT PicoGreen dsDNA kit (Invitrogen). Purified PCR products were diluted to 10 ng/μl and pooled in equal amounts. The pooled amplicons were purified again using Ampure magnetic purification beads (Agencourt) to remove short amplification products.

Illumina reads were merged using PEAR and quality filtered by removing all reads that had at least one base with a q-score lower than 20 [28]. Quality filtered reads were analyzed with the software package QIIME (version 1.9.1) [29]. Sequences were clustered into operational taxonomic units (OTUs) at a 97% identity threshold using an open-reference OTU picking approach with UCLUST [30] against the Greengenes reference database [31] (13_8 release). All sequences that failed to cluster when tested against the Greengenes database were used as input for picking OTUs de novo. Representative sequences for the OTUs were Greengenes reference sequences or cluster seeds and were taxonomically assigned using the Greengenes taxonomy and the Ribosomal Database Project Classifier [32]. Representative OTUs were aligned using PyNAST [33] and used to build a phylogenetic tree with FastTree [34], which was used to calculate α- and β-diversity of samples using Phylogenetic Diversity [35] and UniFrac [36]. Three-dimensional principal coordinates analysis plots were visualized using Emperor [37]. Chimeric sequences were identified with ChimeraSlayer [38] and excluded from all downstream analyses. Similarly, OTUs that could not be aligned with PyNAST, singletons and low abundant OTUs with a relative abundance <0.001% were also excluded. We obtained an average of 68,095.4 ± 13,092.3 sequences/sample (mean ± SD; range 44,539–96,244 sequences/sample); a total of 1,838,576 sequences and 794 OTUs were included in the analyses. To correct for differences in sequencing depth between samples, 44,539 sequences were randomly sub-sampled from each sample and included in the analyses for the estimation of α- and β-diversity.

Sequence data are available at the European Nucleotide Archive (ENA) with accession number PRJEB21676.

### 2.9 Statistical analysis

Data are presented as mean − SEM. Analyses between groups were determined by one-way analysis of variance with ad hoc Bonferroni post tests using GraphPad Prism 7 software. See specific figure legends for more details.

Differences in alpha and beta diversity were tested with the Kruskal–Wallis and Friedman tests adjusted for multiple comparisons. p-Values below 0.05 were considered statistically significant. Clustering of samples by Bray–Curtis and weighted UniFrac distances according to time of sampling and mouse identification number was tested using the adonis function in QIIME with 999 permutations. Wald test with paired design implemented in DESeq2 was used for the analysis of differential abundance of OTUs present in 50% of the samples (in 13 out of the 25 fecal samples) and grouped at genus level [39].

## 3 Results and discussion

### 3.1 Colonization of GF mice produces bi-phasic glucose intolerance

Several studies using colonization of GF mice have focused on medium-long term effect of microbiota colonization (14–28 days, up to 12–15 weeks) on host adiposity and glucose metabolism [40], [41], [42]. Here we evaluated the effect of microbiota–host interaction on adiposity and glucose metabolism during a period of 28 days after colonization using Swiss Webster mice as a model of mild obesity on chow diet in order to study the effect of microbiota on metabolism in obesity to avoid the pro-inflammatory effect of high fat diets [43].

**Figure 1:**
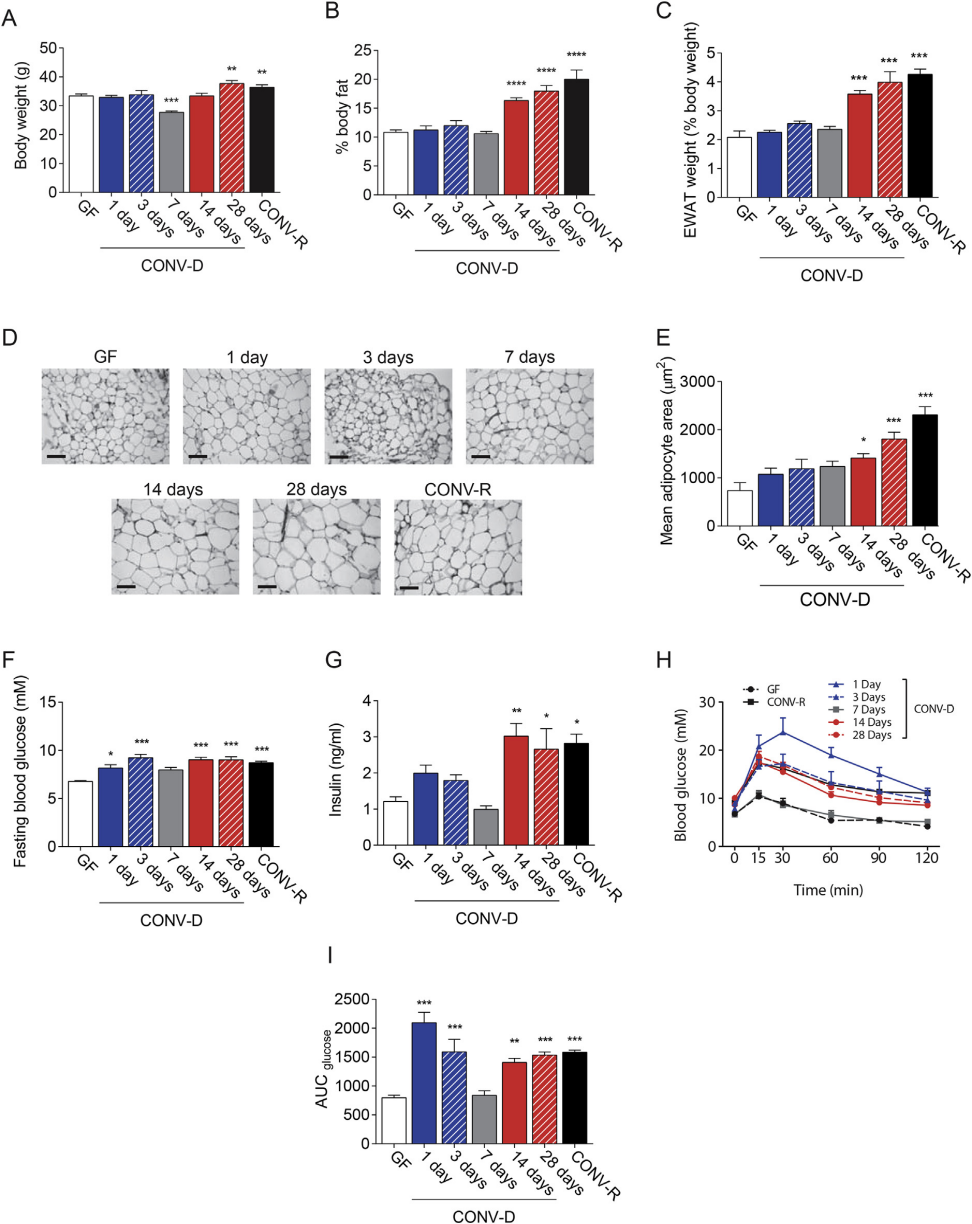
**Colonization of GF mice induces a bi-phasic impairment of glucose metabolism.** Body weight (A), body fat mass percentage (B), and relative epididymal white adipose tissue (EWAT) weight (C) in germ-free (GF), conventionalized (CONV-D), and conventionally raised (CONV-R) mice, 10–15 mice per group. Representative hematoxylin and eosin staining of EWAT (D) (scale bars 100 μm) and mean adipocyte size (E) in GF, CONV-D, and CONV-R mice, 4–10 mice per group. Glucose (F) and insulin (G) levels in GF, CONV-D, and CONV-R mice, 10–15 mice per group. Glucose levels (H) and glucose area under the curve (AUC) (I) during intraperitoneal glucose tolerance in GF, CONV-D, and CONV-R mice, 10–15 mice per group. Data are plotted as mean − SEM. *p < 0.05, **p < 0.01, ***p < 0.001, One-Way ANOVA each group vs GF mice. See also Supplementary Figure 1.

We colonized adult GF mice (conventionalized mice; CONV-D) by oral gavage with cecum content of age- and sex-matched CONV-R mice and analyzed adiposity and glucose metabolism at five different time-points of colonization (1, 3, 7, 14, and 28 days). Body weight was transiently reduced 7 days after colonization (Figure 1A). The reduction in body weight was associated with reduced cecum weight after colonization (Supplementary Figure 1A), a hallmark of the conventionalization process [16]. After 14–28 days of colonization, we observed weight gain (Figure 1A) associated with increased adiposity (Figure 1B), epididymal fat pad (EWAT) weight (Figure 1C), and increased adipocyte size (Figure 1D and E). These findings are likely secondary to microbiota-induced increase of energy harvesting from diet after colonization in the host [16].

Next, we investigated how glucose metabolism was affected during the establishment of the gut microbiota community. Interestingly, we observed two separate phases of increased fasting glucose levels (Figure 1F). A first phase was observed within the first 3 days after colonization (*early phase*), after which, around 7 days post colonization, the levels of fasting glucose decreased and returned to those observed in GF mice. Interestingly, we observed a second phase at 14 and 28 days post colonization (*delayed phase*), coinciding with the increased adiposity and in agreement with previous studies [10], [16]. Insulin levels and HOMA score were significantly increased by colonization in the *delayed phase*, but not in the *early phase* (Figure 1G, Supplementary Figure 1B). In order to characterize glucose metabolism during colonization in more detail, we performed glucose tolerance tests (GTT) at each time point. Similar to fasting glucose levels, we found that glucose tolerance was impaired during the *early phase* and the *delayed phase* compared to GF mice, with the most pronounced attenuation observed 1 day post-colonization and normalization of glucose tolerance similar to GF mice levels around 7 days post colonization (Figure 1H and I). A similar trend was observed when we evaluated insulin levels during GTT (Supplementary Figure 1C). Similar results were observed when insulin tolerance tests (ITT) were performed in a separate cohort of CONV-D, GF, and CONV-R mice (Supplementary Figure 1D and E). Our findings show that microbiota impairs host glucose metabolism in a bi-phasic time dependent fashion during colonization of CONV-D mice. Since the maximum impairment in glucose tolerance was observed at day 1 post colonization, in the absence of significant increase in insulin we propose that this is mediated by dramatic insulin resistance. This is also observed during infections and thus may be attributed to the dramatic load of bacteria and inflammatory molecules [44], [45], [46]. In contrast, the *delayed phase* of glucose impairment is associated with increased adiposity, in agreement with previous publication [10].

### 3.2 Colonization of GF mice is characterized by a bi-phasic systemic inflammatory response

Inflammation is known to affect glucose metabolism, and bacterially produced LPS is one of the mediator of this process [9], [47]. Thus, we evaluated the effect of microbiota colonization on LPS and cytokines serum levels.

**Figure 2:**
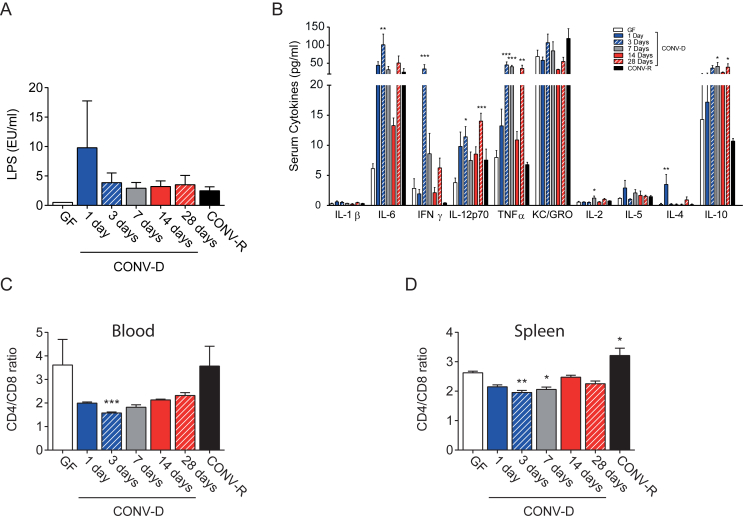
**Colonization of GF mice induces a systemic inflammatory response**. Serum portal LPS levels (A) and peripheral cytokines levels (B) in germ-free (GF), conventionalized (CONV-D), and conventionally raised (CONV-R) mice, 5–10 mice per group. Circulating (C) and spleen resident (D) CD4/CD8 positive lymphocyte ratio, determined by flow cytometry, in GF, CONV-D, and CONV-R mice, 4–10 mice per group. Data are plotted as mean − SEM. *p < 0.05, **p < 0.01, ***p < 0.001, One way ANOVA each group vs GF mice.

In portal blood, we observed a consistent trend towards increased LPS levels in CONV-D mice with a peak at 1 day post colonization (Figure 2A), co-occurring with dramatic impairment of GTT. The increased levels of bacteria-derived inflammatory molecules in the portal vein is likely the result of colonization of an immature gut with impaired barrier function and was mirrored by elevated levels of increased serum levels of pro-inflammatory cytokines, such as IL-6, INFγ, IL-12, and TNFα (Figure 2B). IL-6 and IFNγ reached their highest levels during the *early phase* (within 3 days) while Il-12 and TNFα showed a bi-phasic fluctuation with a first peak of increment in the *early phase*- and a later one in the *delayed phase*. On the other hand, IL-4, which has anti-inflammatory effect promoting the alternative activation of macrophages [48], was transiently increased at time-point 1 day compared to GF mice, while the levels of the anti-inflammatory cytokine IL-10 were slightly increased to a rather constant level from day 3 onwards (Figure 2B). No differences in serum levels of IL-1β, KC/GRO, and Il-5 during colonization. The increased levels of several cytokines in the *early phase* could be responsible *per se* for the *early* phase of glucose impairment and be consequent to the acute microbiota–host interaction, while the increased levels in the *delayed phase* (i.e. TNF alpha, IL-10, and IL-12) can be secondary to the continuous exposure to the microbial components such as LPS (Figure 2A) [49]. These results are consistent with previous published data [22], [50] on colonization of GF, in which cytokine production peaked four days after colonization.

To further characterize the inflammatory response during colonization, we performed flow cytometry analysis of the circulating and spleen-resident T-cells. In CONV-D mice, colonization induced a transient reduction of the CD4/CD8 ratio indicating a systemic inflammatory response during the *early phase* (Figure 2C and D). Taken together, our data indicate that colonization of GF mice results in a systemic inflammatory response, during colonization process. A transient glucose tolerance impairment and increased cytokine release is observed during the first three days of life in humans, associated with colonization of the gut [51], [52], [53].

### 3.3 Colonization of GF mice is characterized by a bi-phasic inflammatory response in liver and adipose tissue

**Figure 3:**
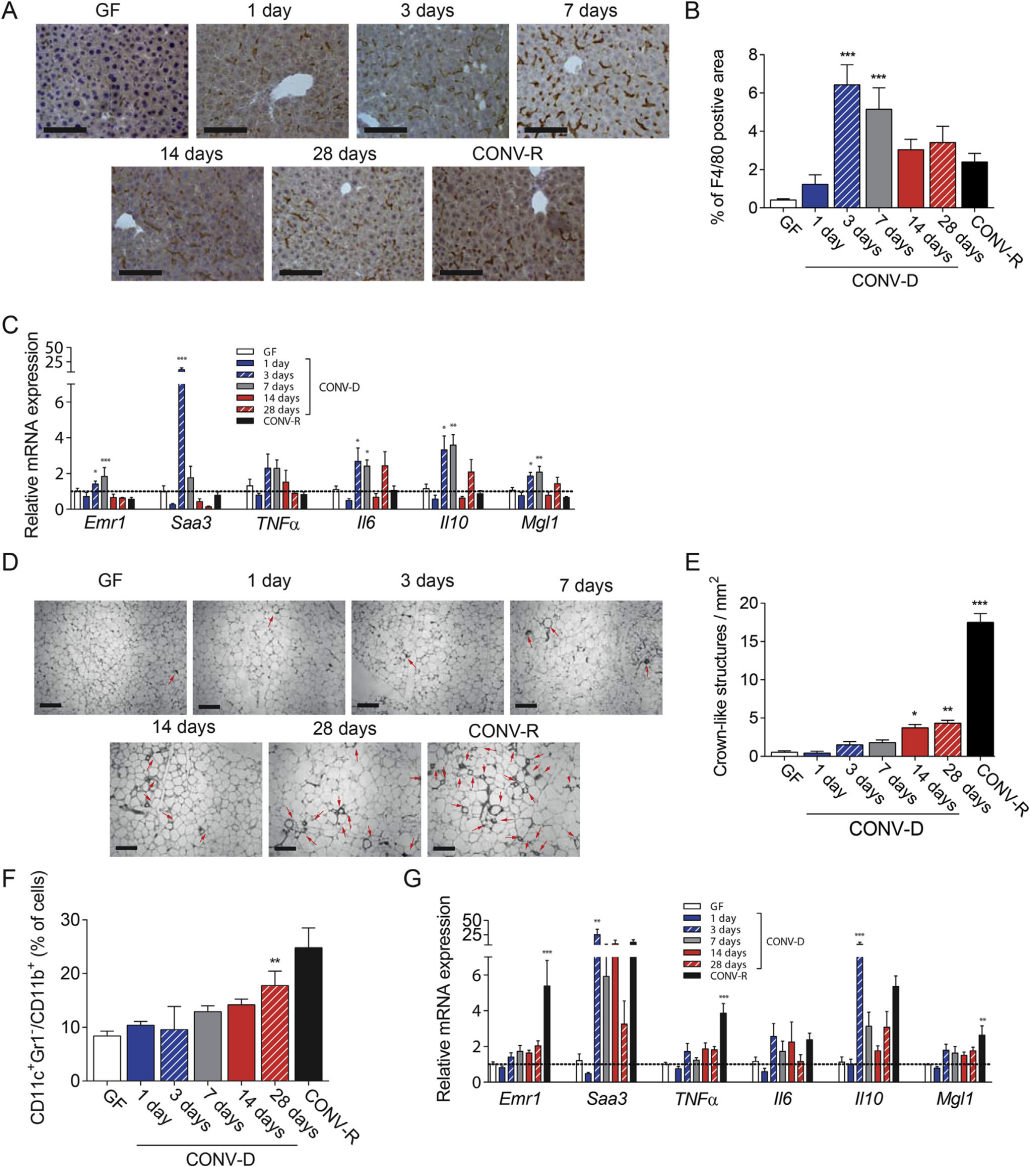
**Colonization of GF mice induces increased inflammatory response in the liver during the *early phase* in the liver and in the EWAT during the *delayed phase***. Representative figures (A) and quantification of F4/80 positive area (B) in the liver, (scale bars 100 μm) in in germ-free (GF), conventionalized (CONV-D) and conventionally raised (CONV-R) mice, 4–10 mice per group. C. Relative mRNA expression in the liver for *Emr1*, *Saa3*, *TNFα*, *Il6*, *Il10*, and *Mgl1* in GF, CONV-D, and CONV-R mice, 5–10 mice per group. Representative figures (D) and quantification (E) of EWAT crown-like structure positive area (scale bars 100 μm), in GF, CONV-D, and CONV-R mice, 4–10 mice per group. Red arrows indicate crow-like structures. F. CD11c^+^, Gr1^−^, CD11b^+^ EWAT resident macrophages, determined by flow cytometry in GF, CONV-D, and CONV-R mice, 4–7 mice per group. G. Relative mRNA expression in the EWAT for *Emr1*, *Saa3*, *TNFα*, *Il6Il10*, and *Mgl1* in GF, CONV-D, and CONV-R mice, 5–10 mice per group. Data are plotted as mean − SEM. *p < 0.05, **p < 0.01, ***p < 0.001 One-Way ANOVA each group vs GF mice. See also Supplementary Figure 2.

Next, we aimed to identify which metabolic tissues (i.e., liver and adipose tissue) were involved in the inflammatory response. We found that the inflammatory response in the *early phase* mainly affects the liver, as shown by the immunostaining for F4/80, a marker of macrophages, which revealed a transient recruitment of these cells during the *early phase* of colonization (Figure 3A and B). This finding was also supported by the qRT-PCR analysis on *Emr1*, the gene encoding for F4/80 protein in mice (Figure 3C). qPCR-RT analysis also showed an increased hepatic expression of some pro- (*Saa3*, *Il6*) and anti- (*Il10*, *Mgl1*) inflammatory markers only in the *early phase* – first week post colonization (Figure 3C). The elevated levels of SAA3, which is an acute phase protein, may be a protective response to the increased influx of microbial products (such as LPS). Colonization did not affect liver weight (data not shown) or liver triglycerides (Supplementary Figure 2).

In EWAT, we immunostained for the macrophage marker MAC-2 to visualize crown-like structures, representing accumulation of macrophages around dead adipocytes, a marker of adipose tissue inflammation associated with impaired glucose metabolism in obesity [54]. We were not able to visualize any significant increase of crown-like structures in the *early phase* whereas an increased accumulation was evident in the *delayed phase* (Figure 3D and E). Moreover, flow cytometry analysis in EWAT for resident pro-inflammatory macrophages (CD11c^+^Gr^−^/CD11b^+^ cells) showed an increased number of these cells only in the *delayed phase* (28 days) of colonization (Figure 3F). qRT-PCR analysis of EWAT for the pan-macrophage marker *Emr1* showed an increased expression in CONV-R compared to GF mice but only a trend towards increased expression in CONV-D mice (Figure 3G). When we analyzed the expression in the EWAT of CONV-D mice of several pro- (*Tnfα*, *Il6*, *Saa3*) and anti-inflammatory *(Mgl1 and Il10)* markers, only *Saa3* and *IL-10* showed significantly increased expression in the *early phase* (3 days) of colonization. These findings indicate that recruitment of inflammatory cells to EWAT occurs in the *delayed phase* of colonization, while the expression of inflammatory markers starts already in *early phase* of colonization. These data are in line with our findings that adiposity is increased 14–28 days after colonization during the *delayed phase* of glucose intolerance (Figure 1B–E). Thus, the impaired glucose metabolism we observed in the *delayed phase* of colonization of GF could be mainly due to the increased whole-body adiposity, adipose tissue inflammation, and adipocyte size. These data are in line with our previous study showing an increased expression of markers for both pro- and anti-inflammatory macrophages in EWAT after colonization of GF mice [10].

Interestingly, despite being the site of a direct contact between the host and the microbiota, the intestine did not show an increased inflammatory response in the *early phase* of colonization. We did not observe any changes in intestinal gene expression for *Emr1* during colonization. Pro- and anti-inflammatory markers such as *Tnfα*, *Il6*, *Il10*, *and Mgl1* were up regulated only after 7–28 days of colonization, while the pro-inflammatory marker *Saa3* showed a single peak of expression only after 7 days of colonization (Supplementary Figure 3). These findings are consistent with our previous results showing that the microbiota–host interaction needs at least one week to induce the expression of genes involved in the immune response towards bacteria in the intestine [55].

Taken together these results indicate that microbiota–host immune system interaction modulates inflammation in different organs in different phases of the colonization process. Specifically, colonization of the naïve GF gut allows influx of pro-inflammatory molecules leading to an acute inflammation with elevated levels of cytokines and production of acute phase proteins, predominantly affecting the liver in the *early phase* of colonization. In contrast, the *delayed phase* is likely a result of increased energy harvest from the diet resulting in increased adiposity in particular due to adipose tissue expansion leading to impaired glucose tolerance. However, we cannot exclude the impact of additional microbially regulated factors such as gastric emptying, vagus nerve tone, and GLP-1 secretion and action [56], [57].

### 3.4 Establishment of a gut microbiota in adult GF mice

To clarify how the microbiota is affected during colonization of GF mice we determined the composition of the fecal microbiota at the same time points as for the measurements of adiposity and glucose metabolism (1, 3, 7, 14 and 28 days in CONV-D mice) using 16S rRNA gene profiling. Species diversity was estimated by the number of observed operational taxonomic units (OTUs; observed species) as well as phylogenetic diversity (PD). We observed that species diversity increased rapidly after colonization and reached a maximum after 28 days (Supplementary Figure 4A and B).

We then used weighted UniFrac and Bray–Curtis indexes to follow the changes in overall microbiota composition at operational taxonomic unit (OTU) level. Principal coordinates analysis (PCoA) showed strong clustering by day of sampling (Supplementary Figure 4C and D; adonis for weighted UniFrac: p = 0.001, r^2^ = 0.69; adonis for Bray–Curtis: p = 0.001, r^2^ = 0.75), and revealed that fecal communities were more similar within time points than within individual mice (adonis analysis showed no clustering by mouse identification number; adonis for weighted UniFrac: p = 0.225, r^2^ = 0.06; adonis for Bray–Curtis: p = 0.376, r^2^ = 0.04). PCoA analysis also showed that the fecal microbiota became increasingly more similar to the inoculated cecal microbiota used to colonize the mice (Supplementary Figure 3E and F), and, in agreement with these results, we found that the number of OTUs shared between fecal samples and the inoculum increased with time, with a successful colonization of about 82% of the inoculated cecal taxa by day 28 (Supplementary Figure 4G and H).

We also investigated the specific changes in relative abundance of microbial genera during colonization and observed significant differences for 30 out of the 35 genera detected in the fecal samples (Supplementary Table 1 and Supplementary Figure 4I), showing that major rearrangements of the fecal microbiota took place at each time point.

Taken together, profiling of the fecal microbiota during colonization of GF mice showed that both species richness and overall microbiota composition changed rapidly during the first week of colonization. As we observed strong clustering of fecal samples by day of sampling, we conclude that specific interactions between colonizing species and gut environment take place at each time point, leading to the selection of a similar gut microbiota in the different mice. We speculate that the activation of specific immune responses and/or the establishment of new intestinal conditions at the different time points might be responsible for the selection of the microbiota, as also previously shown [20], [22].

### 3.5 Re-colonization of antibiotic treated mice is characterized only by a *delayed phase* of glucose impairment

It is noteworthy that GF mice have an immature immune system due to the lack of interaction with bacterial antigens [58]. Thus, the inflammatory response that we observed in the *early phase* could be ascribed to a GF mice immature immune system [58], [59]. In order to investigate how the interaction between microbiota and a normal immune system affects glucose metabolism during colonization, we used CONV-R mice treated with antibiotics for 1 week (Abx mice), which result in mice with a mature immune system and intact gut integrity, but dramatically reduced levels of bacteria. We evaluated adiposity and glucose metabolism before and 1, 3, 7, 14, and 28 days after re-colonization. We referred these mice as conventional re-derived (CONV-RD).

**Figure 4:**
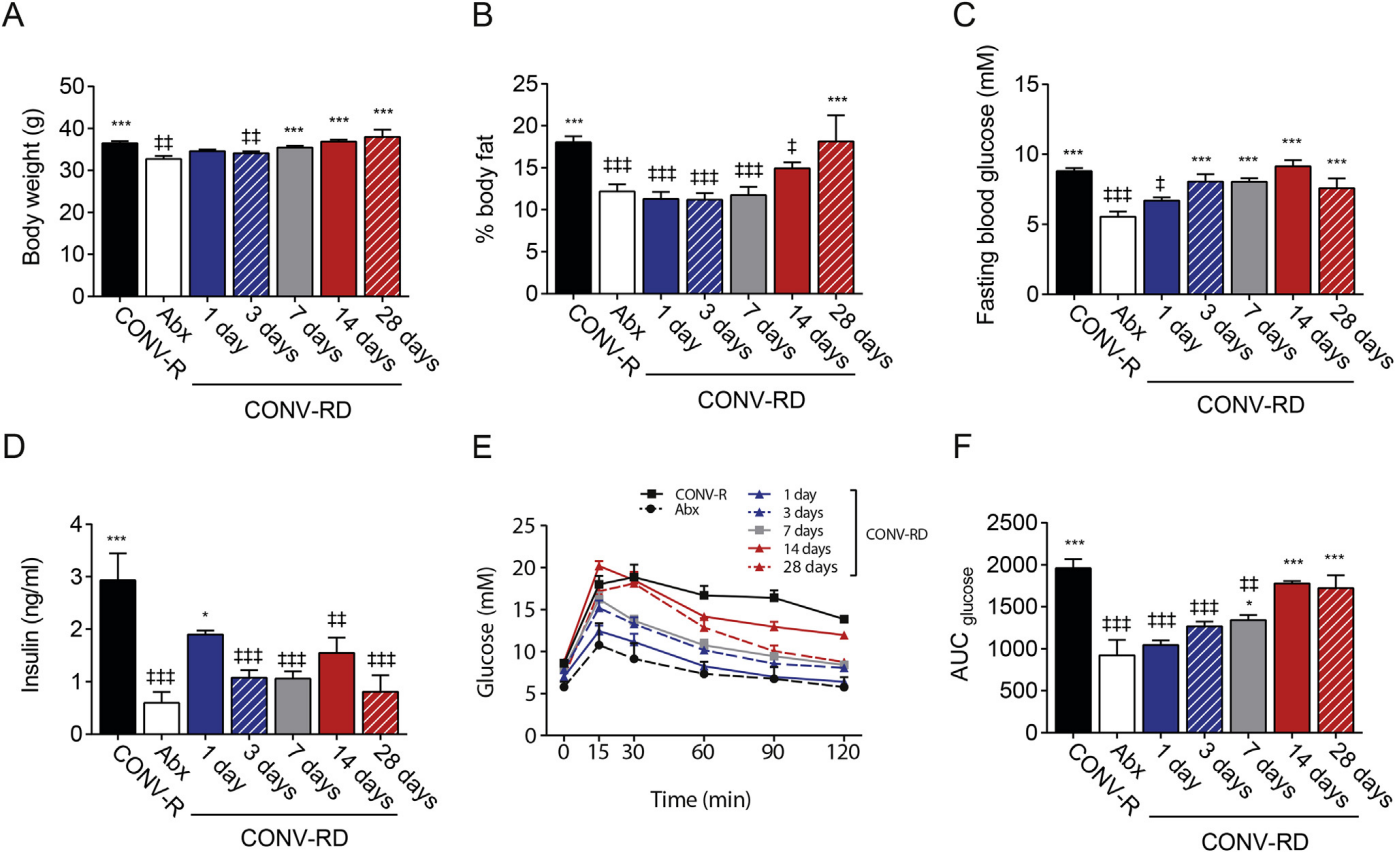
**Colonization of conventionally re-derived mice (CONV-RD) do not induce an *early phase* of glucose impairment**. Body weight (A) and body fat mass percentage (B) in antibiotic treated mice (Abx), conventionally re-derived (CONV-RD) and conventionally raised (CONV-R) mice, 5–13 mice per group. Fasting glucose (C) and insulin (D) levels in Abx, CONV-RD, and CONV-R mice, 5–13 mice per group. Glucose levels (E) and glucose area under the curve (AUC) (F) during intraperitoneal glucose tolerance in Abx, CONV-RD and CONV-R mice, 5–13 mice per group. Data are plotted as mean − SEM. ^∗/‡^p < 0.05, ^‡‡^p < 0.01, ^∗∗∗/‡‡‡^, ^‡^One way ANOVA each group vs Abx mice, ^∗^One way ANOVA each group vs CONV-R mice.

As shown before [18], [19], antibiotic treatment reduced body weight and body fat, which was fully recovered 28 days after re-colonization (Figure 4A and B). CONV-RD mice did not show an *early phase* of impaired fasting glucose levels upon re-colonization, while fasting glucose gradually reached CONV-R mice levels over time (Figure 4C). Insulin levels showed a peak of increase one day after re-colonization (Figure 4D) but did not affect fasting glucose levels. GTT and HOMA showed that CONV-RD mice had a *delayed phase* of glucose impairment but not an *early* phase of glucose impairment (Figure 4E and F, Supplementary Figure 5A and B). Our results suggest a central role of the immune system in the host metabolic control in the early stage of the microbiota colonization process and that the *early phase* of impaired glucose metabolism may be related to the immature gut of GF mice.

Importantly, colonization of GF and Abx mice resulted in a similar increase in adiposity (Figure 1, Figure 4B) after 28 days, suggesting that this is not related to gut integrity or immune system maturation.

## 4 Conclusions

In conclusion, our data reveal that the establishment of the microbial community biphasically modulates glucose tolerance in mice. The *early phase* is likely due to an immature gut, and absent in antibiotic treated CONV-R mice, with resulting increase in intestinal permeability of pro-inflammatory molecules leading to impaired glucose tolerance. The increase in hepatic inflammation may be a result of detoxification of pro-inflammatory molecules such as LPS [60]. The *delayed phase* is induced by increased adiposity, due mainly to increased adiposity and EWAT expansion and inflammation and thus of relevance for type 2 diabetes.

## Appendix A Supplementary data

**Supplementary Figure 1. Colonization of GF mice induces variation in cecum size and glucose impairment during insulin tolerance test**. Percentage of cecum weight (A) and HOMA score (B) 10–15 mice per group in germ-free (GF), conventionalized (CONV-D) and conventionally raised (CONV-R) mice. (C) Insulin levels during intraperitoneal glucose tolerance test, 10–15 mice per group in germ-free (GF), conventionalized (CONV-D) and conventionally raised (CONV-R) mice. Glucose levels (D) and AUC (E) during intraperitoneal insulin tolerance test, 10–15 mice per group in germ-free (GF), conventionalized (CONV-D), and conventionally raised (CONV-R) mice. Data are plotted as mean − SEM. *p < 0.05, **p < 0.01, ***p < 0.001, One-Way ANOVA each group vs GF mice.

**Supplementary Figure 2. Colonization of GF mice do not affect liver triglyceride content**. Hepatic triglyceride content in germ-free (GF), conventionalized (CONV-D), and conventionally raised (CONV-R) mice, 4–7 mice per group. Data are plotted as mean − SEM.

**Supplementary Figure 3. Colonization of GF mice induces an increased inflammatory response in the intestine during the *delayed phase***. A. Relative mRNA expression in the ileum for *Emr1*, *Saa3*, *TNFα*, *Il6Il10*, and *Mgl1* in germ-free (GF), conventionalized (CONV-D), and conventionally raised (CONV-R) mice, 5–10 mice per group. Data are plotted as mean − SEM. *p < 0.05, **p < 0.01, ***p < 0.001, One-Way ANOVA each group vs GF mice.

**Supplementary Figure 4. Changes in gut microbiota during colonization of CONV-D mice**. Feces from conventionalized (CONV-D) mice (n = 5) were collected during 28 days post colonization. Tukey box-plots showing the variation of fecal microbiota species diversity as estimated by the number of (A) observed species and Phylogenetic Diversity (B). ***p < 0.001 for samples collected at day 28 compared to day 1, Friedman test with correction for multiple comparisons. Principal coordinates analysis of gut microbiota composition at OTU level based on weighted UniFrac (C) and Bray–Curtis (D). Each dot represents a fecal microbiota sample colored by day of sampling. The values in brackets indicated on the axes show the percentage of variation explained by the first three principal coordinates (PC). Tukey box-plots showing weighted UniFrac (E) and Bray–Curtis distances (F) calculated to measure the similarity of microbiota composition between the fecal samples collected at the different time points and the inoculum. *p < 0.05, **p < 0.01 for samples compared to day 1 with Kruskal–Wallis and Dunn's multiple comparisons test. Tukey box-plots showing number (G) and percent (H) of OTUs shared between the inoculum and the fecal samples collected at the different time points. *p < 0.05, **p < 0.01 for samples collected at day 14 and 28 compared to day 1, Friedman test with correction for multiple comparisons. See also Supplementary Table 2. I. Heatmap showing changes in abundance of bacterial genera. The color gradient indicates the log2 of median relative abundance at each time point. The 30 genera listed in the heatmap showed a significant change in abundance during the colonization of GF mice (n = 5) as assessed by the Wald test (FDR < 0.05).

**Supplementary Figure 5. HOMA and insulin secretion during colonization of conventionally re-derived mice (CONV-RD)**. HOMA score (A) and insulin levels (B) during intraperitoneal glucose tolerance in antibiotic treated mice (Abx), conventionally re-derived (CONV-RD), and conventionally raised (CONV-R) mice, 5–7 mice per group. Data are plotted as mean − SEM. ^∗/‡^p < 0.05, ^∗∗/‡‡^p < 0.01, ^∗∗∗/‡‡‡^, ^‡^One way ANOVA each group vs Abx mice, ^∗^ One way ANOVA each group vs CONV-R mice.
