# Supplementary figures and images for: Host–microbiota interaction induces bi-phasic inflammation and glucose intolerance in mice
Source: Mol Metab. 2017 Sep 21;6(11):1371–80. doi: 10.1016/j.molmet.2017.08.016 (PMC5681278; doi:10.1016/j.molmet.2017.08.016)

Supplementary figure 1

A

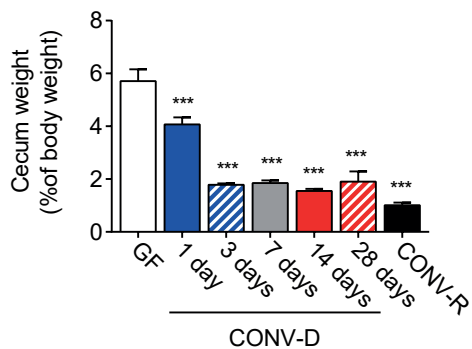

B

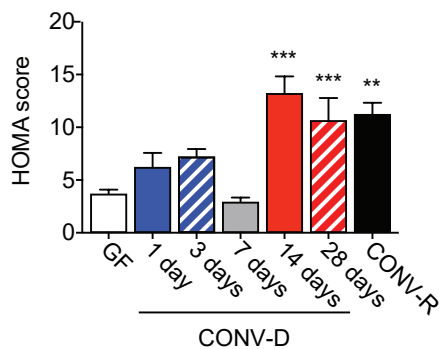

C

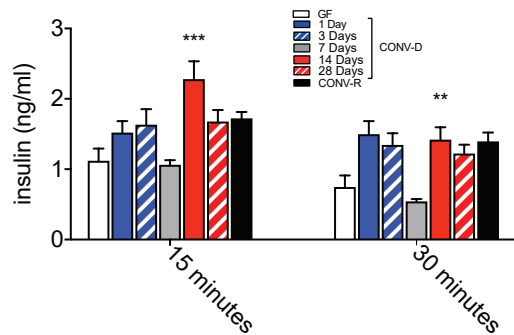

D

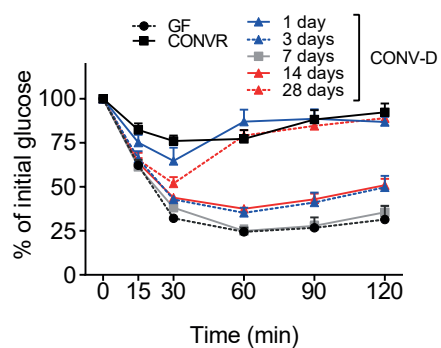

E

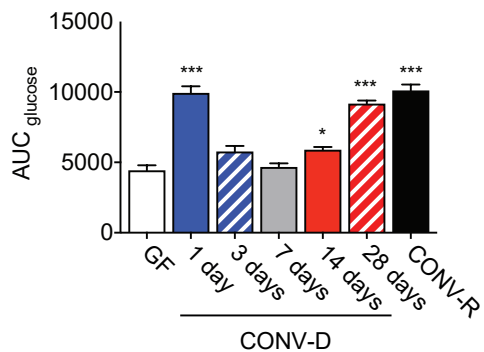

Supplement: Supplementary file 2 — Supplementary Figure 1. Colonization of GF mice induces variation in cecum size and glucose impairment during insulin tolerance test. Percentage of cecum weight (A) and HOMA score (B) 10–15 mice per group in germ-free (GF), conventionalized (CONV-D) and conventionally raised (CONV-R) mice. (C) Insulin levels during intraperitoneal glucose tolerance test, 10–15 mice per group in germ-free (GF), conventionalized (CONV-D) and conventionally raised (CONV-R) mice. Glucose levels (D) and AUC (E) during intraperitoneal insulin tolerance test, 10–15 mice per group in germ-free (GF), conventionalized (CONV-D), and conventionally raised (CONV-R) mice. Data are plotted as mean − SEM. *p < 0.05, **p < 0.01, ***p < 0.001, One-Way ANOVA each group vs GF mice. [file mmc2.pdf]

Supplementary figure 2

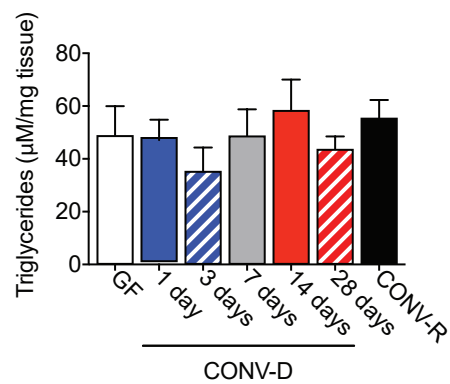

Supplement: Supplementary file 3 — Supplementary Figure 2. Colonization of GF mice do not affect liver triglyceride content. Hepatic triglyceride content in germ-free (GF), conventionalized (CONV-D), and conventionally raised (CONV-R) mice, 4–7 mice per group. Data are plotted as mean − SEM. [file mmc3.pdf]

Supplementary Figure 3

A

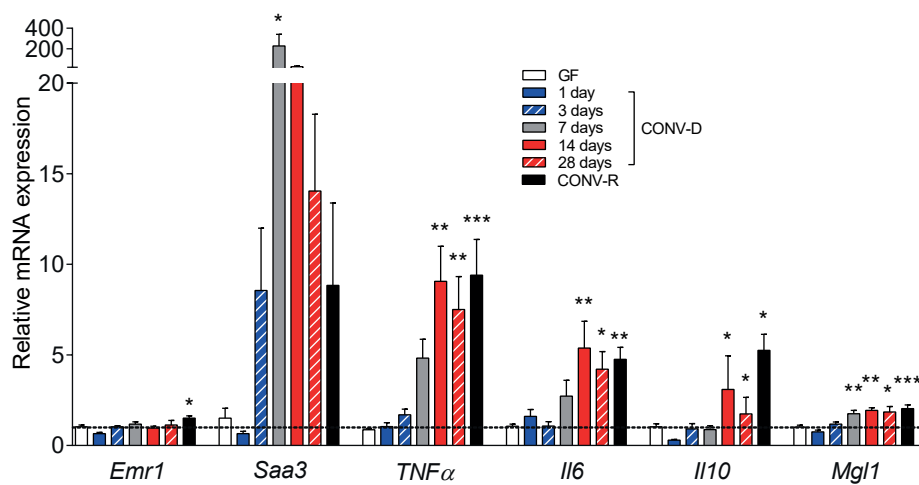

Supplement: Supplementary file 4 — Supplementary Figure 3. Colonization of GF mice induces an increased inflammatory response in the intestine during the delayed phase. A. Relative mRNA expression in the ileum for Emr1, Saa3, TNFα, Il6Il10, and Mgl1 in germ-free (GF), conventionalized (CONV-D), and conventionally raised (CONV-R) mice, 5–10 mice per group. Data are plotted as mean − SEM. *p < 0.05, **p < 0.01, ***p < 0.001, One-Way ANOVA each group vs GF mice. [file mmc4.pdf]

Supplementary Figure 4

A

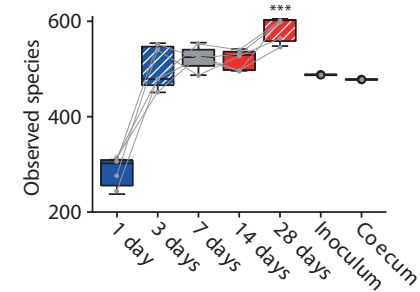

B

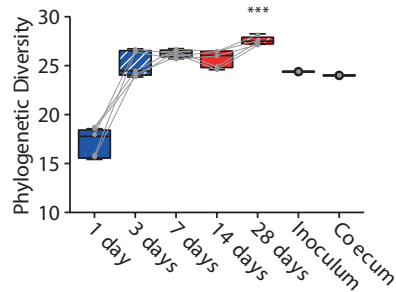

C

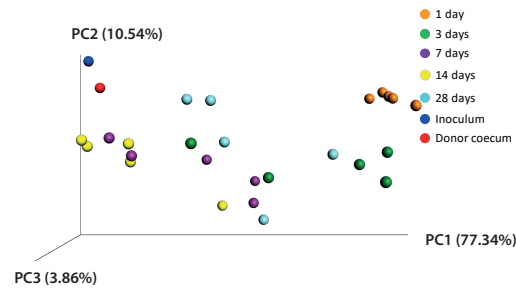

D

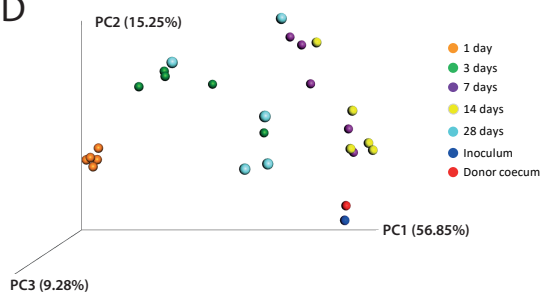

E

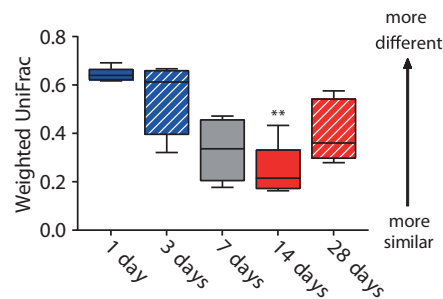

F

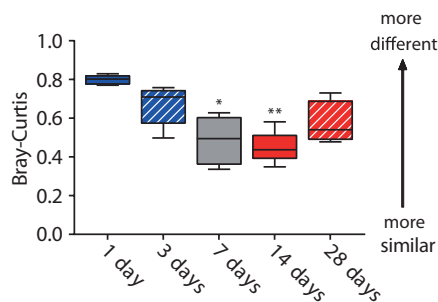

G

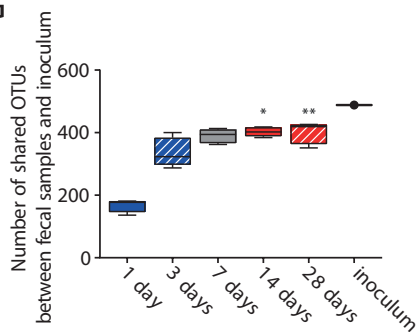

H

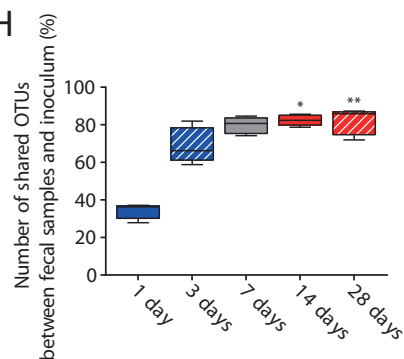

I

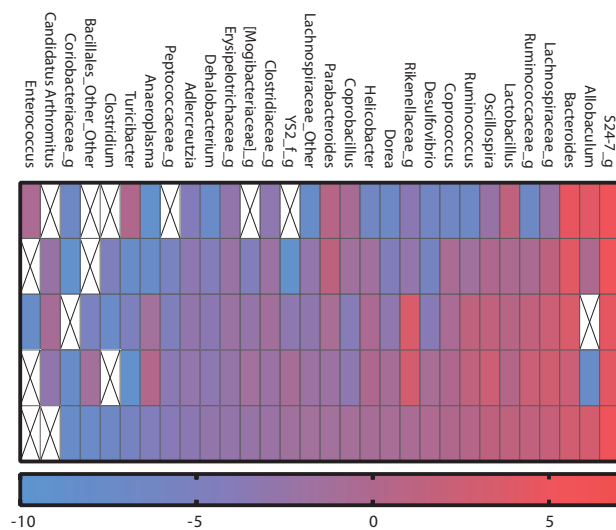

Supplement: Supplementary file 5 — Supplementary Figure 4. Changes in gut microbiota during colonization of CONV-D mice. Feces from conventionalized (CONV-D) mice (n = 5) were collected during 28 days post colonization. Tukey box-plots showing the variation of fecal microbiota species diversity as estimated by the number of (A) observed species and Phylogenetic Diversity (B). ***p < 0.001 for samples collected at day 28 compared to day 1, Friedman test with correction for multiple comparisons. Principal coordinates analysis of gut microbiota composition at OTU level based on weighted UniFrac (C) and Bray–Curtis (D). Each dot represents a fecal microbiota sample colored by day of sampling. The values in brackets indicated on the axes show the percentage of variation explained by the first three principal coordinates (PC). Tukey box-plots showing weighted UniFrac (E) and Bray–Curtis distances (F) calculated to measure the similarity of microbiota composition between the fecal samples collected at the different time points and the inoculum. *p < 0.05, **p < 0.01 for samples compared to day 1 with Kruskal–Wallis and Dunn's multiple comparisons test. Tukey box-plots showing number (G) and percent (H) of OTUs shared between the inoculum and the fecal samples collected at the different time points. *p < 0.05, **p < 0.01 for samples collected at day 14 and 28 compared to day 1, Friedman test with correction for multiple comparisons. See also Supplementary Table 2. I. Heatmap showing changes in abundance of bacterial genera. The color gradient indicates the log2 of median relative abundance at each time point. The 30 genera listed in the heatmap showed a significant change in abundance during the colonization of GF mice (n = 5) as assessed by the Wald test (FDR < 0.05). [file mmc5.pdf]

A

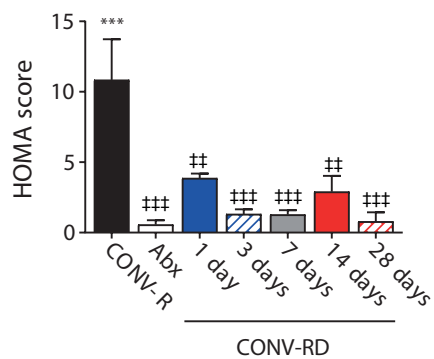

B

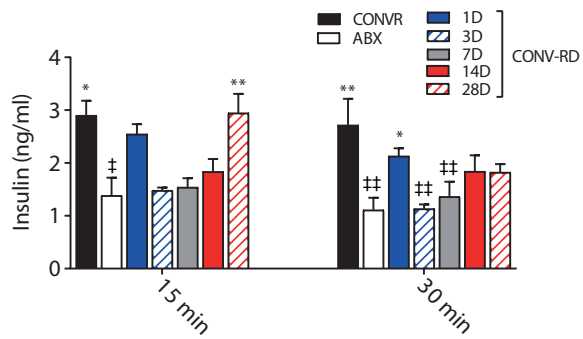

Supplement: Supplementary file 6 — Supplementary Figure 5. HOMA and insulin secretion during colonization of conventionally re-derived mice (CONV-RD). HOMA score (A) and insulin levels (B) during intraperitoneal glucose tolerance in antibiotic treated mice (Abx), conventionally re-derived (CONV-RD), and conventionally raised (CONV-R) mice, 5–7 mice per group. Data are plotted as mean − SEM. ∗/‡p < 0.05, ∗∗/‡‡p < 0.01, ∗∗∗/‡‡‡, ‡One way ANOVA each group vs Abx mice, ∗ One way ANOVA each group vs CONV-R mice. [file mmc6.pdf]
